# Obstructive Uropathy Secondary to Missed Acute Appendicitis

**DOI:** 10.1155/2016/4641974

**Published:** 2016-10-12

**Authors:** Mahir Gachabayov

**Affiliations:** Vladimir City Clinical Hospital of Emergency Medicine, Stavrovskaya Street, 6-73, Vladimir 600022, Russia

## Abstract

Hydronephrosis is a rare complication of acute appendicitis. We present a case of missed appendicitis in a 52-year-old female which presented as a right-sided hydronephrosis. 2 days after admission to the Department of Urology CT revealed acute appendicitis for what open appendectomy was performed. Acute appendicitis can lead to obstructive uropathy by periappendiceal inflammation due to adjacency. Urologists, surgeons, and emergency physicians should be aware of this rare complication of atypical acute appendicitis.

## 1. Introduction

Acute appendicitis is the most commonly encountered surgical abdominal emergency with the estimated lifetime risk of 8.6% for males and 6.7% for females [1]. In surgical society acute appendicitis is usually considered a “simple” disease. Despite this fact, despite the high prevalence and large number of publications there are still challenging cases. Negative appendectomy rate has been shown to be up to 15.3% with the highest incidence among women, children younger than 5 years old, and elderly people older than 65 years old [2]. The frequency of misdiagnosed appendicitis has not changed with the introduction of computed tomography, ultrasonography, and laparoscopy or has the frequency of perforation decreased [3]. Some authors were right to have named acute appendicitis a “chameleon-like” disease. A “classical presentation” of acute appendicitis has been shown to be only 6% of patients with suspected appendicitis [4]. Atypical clinical presentation can be encountered in some populations more often, such as elderly or pregnant patients, and can depend on the anatomic features of vermiform appendix. The complication rate of acute appendicitis is 4–15%, and the overall mortality rate is 0.2–0.8% [5]. Hydronephrosis is a rare complication of acute appendicitis.

## 2. Case Presentation

**Figure 1:**
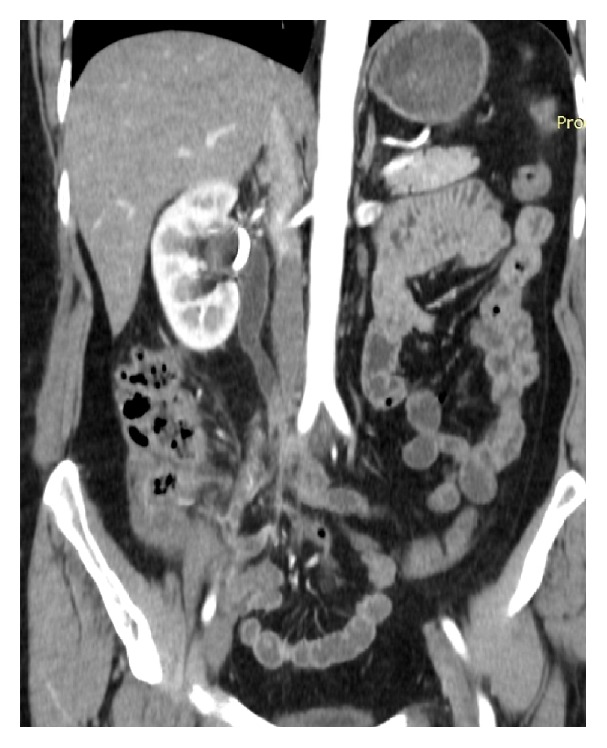
Hydronephrosis on abdominal CT, coronal plane.

**Figure 2:**
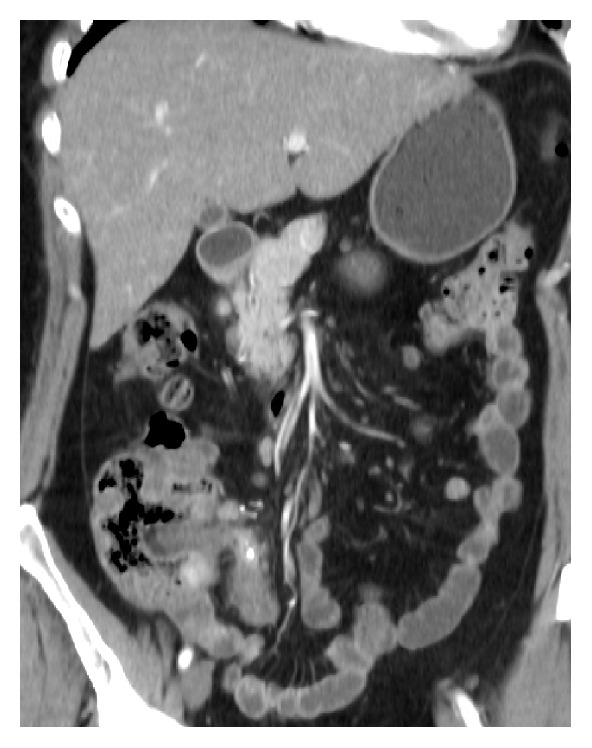
Appendicitis on abdominal CT, coronal plane.

**Figure 3:**
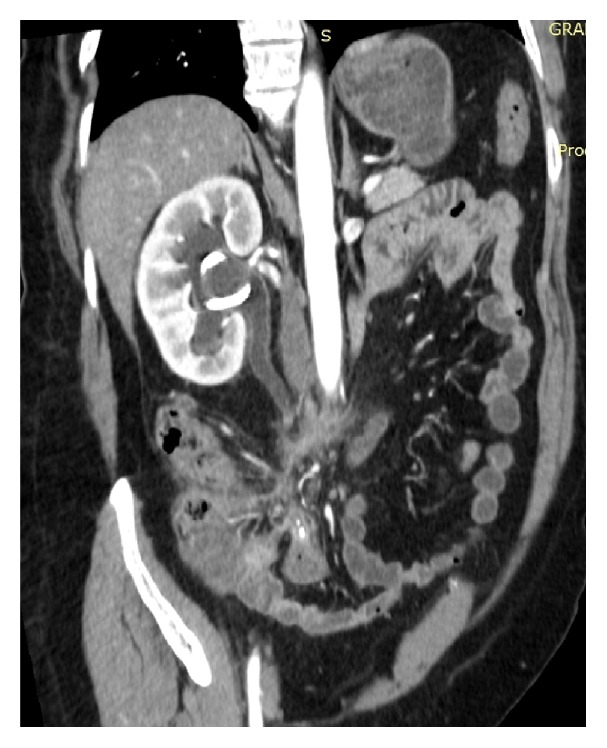
Acute appendicitis and periappendiceal inflammation obstructing right ureter on abdominal CT, oblique plane.

A 52-year-old female patient was admitted to Vladimir City Clinical Hospital of Emergency Medicine with a 4-day-history of right flank pain and dysuria. Her past medical history was insignificant. She did not have any episodes of renal colic before. On admission her body temperature was 38.2°C, heart rate was 108 bpm, BP was 110/70 mmHg, Hb was 13.2 mg/dL, WBC was 18,000/*μ*L, and band neutrophils were 12%. The patient noticed that this was the first such episode in her life, she also noticed that the pain started in periumbilical region, and she had nausea and anorexia. Abdominal USG revealed right-sided hydronephrosis without evidence of nephrolithiasis. On physical exam tenderness on right mesogastrium with no rebound tenderness was noted. The patient was hospitalized to the Department of Urology where percutaneous nephrostomy was performed and wide spectrum antibiotics were started. Despite the treatment the patient remained septic. 2 days after admission the patient was consulted by a surgeon. Both the Alvarado and the Anderson scores appeared to be 7 and the Adult Appendicitis score appeared to be 16. Abdominal CT was performed which revealed acute appendicitis to be the cause of ureteric obstruction (Figures 1, 2, and 3). The patient underwent open appendectomy, and retrocaecal gangrenous appendix was removed. Postoperatively, the patient recovered without any complications and was discharged on 8th postoperative day. Before discharge abdominal USG was performed on which obstructive uropathy was resolved. On follow-up after 2 months the patient was well.

## 3. Discussion

Starting with Littre A., several cases have been published on hydronephrosis as a complication of acute appendicitis [6]. The main pathogenetic factor is the influence of periappendiceal inflammation due to adjacency. Therefore the most commonly affected site is right ureter [7]. However, bilateral hydronephrosis has also been shown to be a possible complication of acute appendicitis [8].

The clinical presentation of acute appendicitis is very varicolored and is influenced by several factors including the location of vermiform appendix or the complication which occurred. Therefore there is no pathognomonic sign and symptom specific for acute appendicitis. To improve the chances of accurate diagnosis several tools have been developed, such as diagnostic scales. Alvarado score, Andersson score, and Adult Appendicitis score have shown to improve results [9–11]. In our patient the clinical presentation was not a “classical presentation” of acute appendicitis, neither was it a “classical presentation” of typical causes of obstructive uropathy. The index of suspicion of acute appendicitis seems to be low during admission. The strategy on admission to the Department of Urology was to relieve ureteric obstruction followed by more detailed examination after relief. The diagnostic scores were evaluated 2 days after admission which showed at least medium suspicion of acute appendicitis.

Another very important point in the management of acute appendicitis is imaging. In most cases USG plays a role of first-line imaging modality in both urological and surgical practice. The sensitivity and the specificity of USG are 86.7% and 90.0%, respectively. Yu et al. have shown that the advantages of USG for acute appendicitis were mainly found in young and male patients who are highly clinically suggestive [12]. In our case USG failed to diagnose acute appendicitis and to reveal the cause of hydronephrosis as well. In a review of CT versus USG (graded compression) in the diagnosis of acute appendicitis the mean respective sensitivities of CT and ultrasound emerged to be 91% and 78% and the respective specificities 90% and 83% [13]. In our case CT was accurate in the diagnosis of appendicitis and secondary hydronephrosis as well.

The treatment of acute appendicitis is surgery. Laparoscopic appendectomy is performed increasingly more all around the world. Laparoscopic appendectomy has shown shorter hospital stay, better recovery, and cosmesis but longer operative time and more postoperative intraperitoneal complications as compared to open approached. Therefore patient selection is important in both open and laparoscopic appendectomy [5].

To conclude, surgeons, urologists, and emergency physicians should be aware of hydronephrosis as a complication of atypical acute appendicitis, especially if the presentation of obstructive uropathy is also “nonclassical.” In suspicious cases appendicitis diagnostic scores and radiology (especially CT) should be utilized.
